# Biomarkers of HFpEF: Natriuretic Peptides, High-Sensitivity Troponins and Beyond

**DOI:** 10.3390/jcdd9080256

**Published:** 2022-08-10

**Authors:** Paolo Morfino, Alberto Aimo, Vincenzo Castiglione, Giuseppe Vergaro, Michele Emdin, Aldo Clerico

**Affiliations:** 1 Interdisciplinary Center of Health Sciences, Scuola Superiore Sant’Anna, 56127 Pisa, Italy; 2 Cardiology Division, Fondazione Toscana Gabriele Monasterio, 56127 Pisa, Italy

**Keywords:** biomarkers, heart failure, preserved ejection fraction, HFpEF

## Abstract

Heart failure (HF) is a significant cause of morbidity and mortality worldwide. HF with preserved ejection fraction (HFpEF) is a complex syndrome, often participated by several cardiac and extracardiac conditions, including chronic kidney disease, pulmonary disease, anaemia and advanced age. Circulating biomarkers reflecting pathophysiological pathways involved in HFpEF development and progression may assist clinicians in early diagnosis and management of this condition. Natriuretic peptides (NPs) are cardioprotective hormones released by cardiomyocytes in response to pressure or volume overload and in response to activation of neuro-endocrine-immune system. The relevance of B-type NP (BNP) and N-terminal pro-B-type NP (NT-proBNP) for diagnosis and risk stratification has been extensively demonstrated, and these biomarkers are emerging tools for population screening and as guides to the start of treatment in subclinical HF. On the contrary, conflicting evidence exists on the value of NPs to guide HF therapy. Among the other biomarkers, high-sensitivity troponins and soluble suppression of tumorigenesis-2 are the most promising biomarkers for risk stratification, predicting outcome independently from NPs. In this review, some novel biomarkers are being tested in such clinical scenario, more tightly linked to specific pathophysiological processes of cardiac damage.

## 1. Background

Heart failure (HF) is a progressive condition in which the heart muscle is not able to pump enough blood to meet the needs of the body. The prevalence of HF is in 1–2% of adults in industrialized countries and is increasing with population ageing. HF then represents one of the major public health problems [1]. Heart failure with preserved ejection fraction (HFpEF) is a clinical syndrome in which patients have clinical features of HF in the presence of normal or near-normal left ventricular ejection fraction (LVEF), with LV not filling adequately because of diastolic dysfunction. HFpEF accounts for more than half of HF cases [2,3]. HFpEF is currently identified by a LVEF ≥ 50%, although different definitions of “preserved” EF have been employed in previous studies, with LVEF cut-offs ranging from 40% to 55% [4]. HFpEF was initially considered as a cardiac disorder characterized by diastolic dysfunction, cardiomyocyte hypertrophy and myocardial fibrosis. However, extra-cardiac mechanisms also have a crucial role to play in the pathophysiology of HFpEF, leading it to be rebranded as a multisystem disorder [5,6]. Indeed, HFpEF is frequently associated with non-cardiovascular comorbidities (e.g., chronic kidney disease, anemia, chronic obstructive pulmonary disease). All these diseases, as well as advanced age, promote a mild chronic inflammatory state. Myocardial microvascular inflammation, mediated by pro-inflammatory cytokines, leads to activation of endothelial cells, which highly express adhesion molecules that trigger monocyte migration from the bloodstream into the myocardium and their differentiation into macrophages. This vicious circle leads to a state called “endothelial dysfunction”, which contributes to fibrosis and progressive diastolic dysfunction [7,8]. Conversely, HF with reduced ejection fraction (HFrEF; LVEF ≤ 40%) is mainly characterized by systolic dysfunction as a consequence of a direct heart damage, such as an acute coronary syndrome, a cardiomyopathy or a valve disease. Therefore, the pathophysiology of HFpEF is multifactorial, whereas HFrEF is mostly associated with a neuroendocrine-based dysregulation of cardiovascular systems [9].

The risk of all-cause death is comparable in HFpEF and HF with mildly reduced EF (HFmrEF; LVEF 41–49%) and lower than in HFrEF, while the risk of death or HF hospitalization is lower for HFpEF than HFrEF or HFmrEF [10,11]. While several pharmacological and non-pharmacological approaches have been demonstrated to improve survival in HFrEF, only few interventions have proven able to modify the clinical course of HFpEF, possibly due to a consistent phenotypic variability and to the enrolment of heterogeneous populations in large clinical trials [4,12]. However, the recent EMPEROR-Preserved (EMPagliflozin outcome trial in Patients With chronic heart Failure With Preserved Ejection Fraction) trial has demonstrated improved outcomes in patients with HF and LVEF > 40% with the sodium-glucose cotransporter 2 inhibitor (SGLT2i) empagliflozin as compared to the placebo. There was no signal of a differential effect in the subgroups with or without diabetes as well as in patients with LVEF below and above 50% [13,14]. The results of the DELIVER (Dapagliflozin Evaluation to Improve the LIVEs of Patients With Preserved Ejection Fraction Heart Failure) trial (NCT03619213), a phase III trial enrolling HFpEF and HFmrEF patients and testing dapagliflozin versus placebo, are expected in the near future [15].

**Figure 1:**
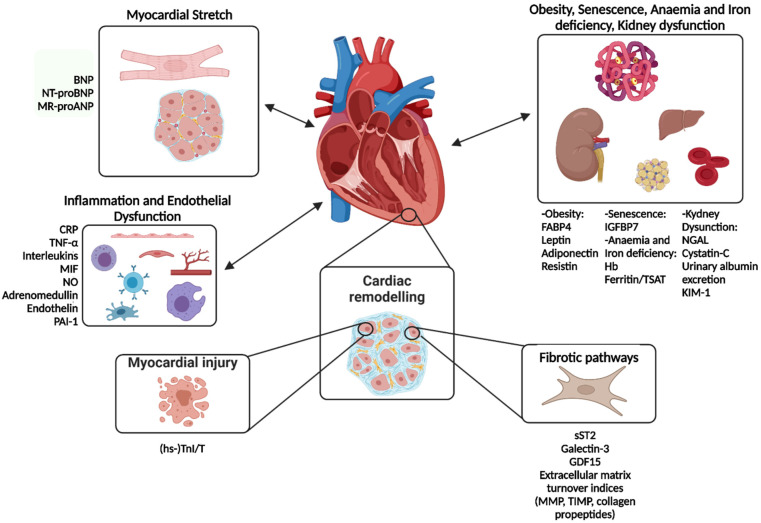
Main pathophysiological pathways involved in HFpEF and their most representative biomarkers. BNP: B-type natriuretic peptide, CRP: C-reactive protein, FABP4: fatty-acid-binding protein 4, GDF-15: growth differentiation factor, Hb: Hemoglobin, hs-TnI/T: high sensitivity-troponin I/T, IGFBP7: insulin-like growth factor-binding protein 7, KIM-1: kidney injury molecule 1, MIF: macrophage migration inhibitory factor, MMP: matrix metalloproteases, MR-proANP: mid-regional pro-atrial natriuretic peptide, NGAL: neutrophil gelatinase-associated lipocalin, NO: nitric oxide, NT-proBNP: N-terminal pro-B-type natriuretic peptide, PAI-1: plasminogen activator inhibitor 1, sST2: soluble suppression of tumorigenesis-2, TIMP: tissue inhibitor of metalloproteinase, TNF-α: tumor necrosis factor alpha, TSAT: transferrin saturation. Modified with permission from Castiglione et al., 2022 [9].

Circulating biomarkers are possible tools to identify patients at higher risk, those with activation of specific pathways of organ damage and, possibly, to provide support for therapy optimization. Both conventional and novel biomarkers may be helpful to predict future disease development, for diagnosis and risk stratification, as well as inclusion and exclusion criteria for clinical trials [16]. A broader discussion on HF biomarkers can be found in a dedicated review paper [9]. Among the many biomarkers related to different aspects of HF pathogenesis (Figure 1), some have received particular attention in the setting of HFpEF and will be discussed in a greater detail here. We will discuss the evidence from trials including at least a subgroup of patients with LVEF ≥ 50%, as it is virtually impossible to analyze separately the evidence on patients with LVEF ≥ 50%, particularly when it comes to biomarker studies [17,18].

## 2. Natriuretic Peptides

B-type natriuretic peptide (BNP) and the N-terminal fragment of proBNP (NT-proBNP) are produced from the cleavage of their 108-aminoacid precursor proBNP by proprotein convertases, such as corin and furin. The biologically active BNP is degraded by several peptidases, such as dipeptidyl peptidase IV and neutral endopeptidases (NEP or neprylisin) [19,20,21]. BNP is produced by ventricular myocytes in response to any kind of damage to the cardiovascular system, including increased myocardial wall stress, and plays a major role in HF pathophysiology, by counteracting the detrimental effects of renin-angiotensin-aldosterone system (RAAS) and sympathetic nervous system activation through their diuretic, natriuretic, vasodilator and anti-hypertrophic properties [22].

BNP and NT-proBNP are crucial biomarkers for the diagnosis of HFrEF, with a less established role for risk stratification and management [4,23]. Nonetheless, their clinical value has been studied across the whole spectrum of LV systolic function. Circulating levels of NPs are increased in patients with HFpEF and mirror the severity of cardiac morphological and functional abnormalities, such as LV hypertrophy, fibrosis and diastolic dysfunction [24,25]. Therefore, their measurement is a central element in the diagnostic algorithm for HF [4,23]. NP levels are less elevated in HFpEF than HFrEF, but no single cut-off value has been shown to accurately differentiate the two conditions [26,27]. A meta-analysis of 51 studies reported that NPs have reasonable diagnostic performance in the detection of HFpEF in a chronic setting (area under the receiver operating characteristics curve [AUC] 0.80; 95% CI 0.73–0.87) [28].

Comorbidities influence NP circulating levels in both HFpEF and HFrEF, which is of great clinical importance, given the higher prevalence of non-cardiac conditions in HFpEF. Several conditions are associated with higher NPs, including chronic obstructive pulmonary disease (COPD), atrial fibrillation (AF), kidney disease, diabetic ketosis, while NP levels may be significantly reduced in obese patients [29,30,31]. Furthermore, increased age is associated with higher NP concentrations [29,32].

NP elevation has been used in many trials as an inclusion criterion to improve the diagnostic specificity and for risk enrichment [33,34]. BNP and NT-proBNP have also been tested as tools for risk stratification. The prognostic performance of BNP in HFpEF is similar to that in HFrEF, since the rates of death and HF related hospitalization are similar to those of patients with impairment of systolic function for any given level of BNP [27]. In the Irbesartan in Patients with Heart Failure and Preserved Systolic Function Study (I-PRESERVE), which enrolled 4128 patients with HF and an LVEF ≥ 45% for a mean follow-up of 49.5 months, NT-proBNP above the median value of 339 ng/L was independently associated with an increased risk of the primary composite endpoint of all-cause death and cardiovascular hospitalization in patients with LVEF ≥ 45% [35].

In a sub-analysis of the I-PRESERVE trial including 2162 patients, Jhund et al. have investigated the association between changes in NT-proBNP over a 6-month follow-up and clinical outcomes (cardiovascular death or HF hospitalization; all-cause death, HF death or HF hospitalization). Changes in NT-proBNP were associated with the risk of clinical outcomes, and particularly with HF-related outcomes. A 1000 ng/L elevation in NT-proBNP over 6 months was associated with a 2-fold higher risk of cardiovascular death or HF hospitalization (HR 2.01, 95% CI 1.50–2.61) [36].

The prognostic role of NT-proBNP has been also evaluated in a recent analysis of the EMPEROR-Preserved trial, which enrolled and randomized 5988 patients with a LVEF > 40% and NYHA class II-IV to receive empaglifozin or a placebo [37]. 5986 (99.9%) participants had available baseline NT-proBNP measurements, with an overall median baseline NT-proBNP level of 974 ng/L (Q1 and Q3 at 499 and 1731 ng/L, respectively). Patients with higher NT-proBNP concentrations were older and showed a more severe degree of HF, including lower LVEF, worse clinical manifestations and health status measured by KCCQ score. Moreover, an increase in baseline NT-proBNP across quartiles was reflected by an enhanced risk of cardiovascular (CV) death, >4-fold higher in the placebo group compared with the highest quartile, and HF hospitalization, 5-fold higher total number of hospitalizations in the placebo group compared with the highest quartile. The increase in NT-proBNP level from baseline to 12 week was associated with risk for CV death both in the placebo (HR: 1.88, 95% CI: 1.57–2.26) and empagliflozin (HR: 1.57, 95% CI: 1.30–1.90) group. Treatment with empagliflozin reduced clinical outcomes across NT-proBNP quartiles without interaction with baseline NT-proBNP and contributing to mildly reduce NT-proBNP levels [37].

Even in patients with acute HF and preserved EF, NT-proBNP was a strong predictor of all-cause mortality [38,39,40]. In a study conducted on 205 patients with HFpEF hospitalized for acute HF, after a mean follow up of 28 ± 10 months, discharge NT-proBNP ≥ 1500 ng/L (HR: 5.23, CI 95%: 2.87–17.8, *p* < 0.001) and ≥50% NT-proBNP reduction between admission and discharge (HR: 0.62, CI 95%: 0.25–0.79, *p* = 0.019) were independent predictors of death and rehospitalization for HF. Moreover, the combination of E/e’ and NT-proBNP values at discharge significantly improved the prognostic ability compared to each variable analyzed independently (AUC, NT-proBNP at discharge: 0.80; E/e’ at discharge: 0.77; E/e’ + NT-proBNP: 0.88; *p* < 0.01) [41]. Another study explored the prognostic significance of NT-proBNP levels in patients hospitalized for acute HF with preserved versus reduced EF. Notably, discharge NT-proBNP concentrations predicted clinical outcomes similarly in HFpEF and HFrEF. In a cohort of patients with HFpEF (*n* = 283) compared to those with HFrEF (*n* = 776) followed up for 6 months, multivariable adjusted Cox regression analysis reported that for any 2.7-factor increase in NT-proBNP levels, the HR for mortality was 2.14 for HFpEF (95% CI 1.48 to 3.09) and 1.96 for HFrEF (95% CI 1.60 to 2.40). Furthermore, prognostically relevant comorbidities were more often present in patients with HFpEF than patients with HFrEF, but only in low (≤3000 ng/L) and not in high (>3000 ng/L) NT-proBNP discharge categories [40].

Guideline-recommended HF therapies reduce NP levels [42]. The use of NPs to guide HF therapy is still controversial, even though some metanalyses showed that a NP-guided treatment is associated with lower rates of all-cause mortality and HF hospitalization [43,44,45], but some uncertainty is reported in other systemic reviews [46,47]. A few studies have explored the effectiveness of a NP-guided therapy in HFpEF. Most notably, patients with LVEF > 45% randomized to medical therapy titrated to reduce symptoms to NYHA ≤ II presented a better 18-month outcome compared to those whose treatment also pointed at a reduction in NT-proBNP below the inclusion thresholds (>400 ng/L or >800 ng/L according to the age) [48]. Overall, further evaluation is warranted to better understand the differences between a NP-guided therapy and a clinically-guided therapy, and for the potential use of NPs in the follow up of HF patients.

As for other NPs, the measurement of circulating atrial NP (ANP) is complicated due to its short half-life (2–5 min) related to the rapid cleavage by neprilysin, insulin-degrading enzyme and natriuretic peptide receptor-C. ANP precursor (proANP), which is stoichiometrically equimolar to ANP, has a longer half-life and it is more easily measurable by searching its mid-regional portion (MR-proANP) [9,49]. MR-proANP has been firstly examined for diagnosis in the Biomarkers in the Acute Heart Failure trial (BACH), in which it revealed great diagnostic ability in acute decompensated HF (cut-off point of ≥120 pmol/L had a sensitivity of 97%, specificity of 60% with accuracy of 74%) [50]. The use of MR-proANP as a biomarker has not yet been extensively investigated in HFpEF, but recent studies highlight its prognostic value. In a study population of 806 subjects with type 2 diabetes (T2D) from the Tousand&2 Study including 141 (17.5%) patients with HFpEF, 646 controls without HF and 19 patients with HFrEF, authors evaluated the association between cardiovascular events and MR-proANP, during a median follow up of 4.8 years. MR-proANP level was associated with a higher risk of incident cardiovascular events (multivariable model HR: 2.56, 95% CI 1.64–4.00) in patients with HFpEF and high MR-proANP, while patients with HFpEF and a low MR-proANP did not show a different risk for incident cardiovascular events compared to patients without HF (multivariable model HR: 2.18, 95% CI 0.78–6.14) [51]. Similar results were obtained on a cohort of 143 patients, including 57 controls without HF, 43 patients with HFpEF and 43 with HFrEF. MR-proANP was associated with the endpoint of HF hospitalization or death in HFpEF (HR adjusted for age, sex, and body mass index [BMI] 1.61, 95% CI 1.07–2.32) [52].

## 3. Troponins

Cardiac troponins are released from intracellular space to the bloodstream following alterations in membrane properties. The release of myocardial troponins may not require myocardial cell death, but the extrusion of proteins from reversibly injured cardiomyocytes may occur during transient increases in cell permeability due to cell wounds [53,54]. In patients with HF, increased levels of troponin generally correlate with HF severity, especially in the acute setting, but the elevation of circulating troponins has been reported also in chronic HF, possibly due to mechanisms such as inflammation, neurohormonal activation, myocardial stretch, hypoxia, cytotoxicity [55].

Santhanakrishnan et al. have shown that hs-TnT was higher in HFrEF than in HFpEF (*p* < 0.04), after adjustment for age, sex and other clinical covariates (e.g., estimated glomerular filtration rate (eGFR), diabetes, hypertension, coronary artery disease, AF, and HF therapies) in 50 patients with HFpEF, 51 with HFrEF and 50 controls without HF [56]. Other studies on larger cohorts confirmed the higher hs-TnT levels in HFrEF and HFmrEF patients compared to HFpEF patients [57,58]. For example, the Trial of Intensified vs. Standard Medical Therapy in Elderly Patients With Congestive Heart Failure (TIME-CHF) study confirmed that patients with LVEF ≥ 50% have lower hs-TnT than those with LVEF ≤ 40% (27.7 [16.8–48.0] vs. 32.4 [19.2–59.0] ng/L, *p* = 0.03) [57]. Both hs-cTnT and hs-cTnI levels are increased in chronic HFpEF and show a stronger association with poorer outcomes in men (HR 3.33; 95% CI 1.82–6.09) than in women (HR 1.35; 95% CI 0.94–1.93), while there were no significant differences in HFrEF [59]. The mechanism of this sex-related difference in the prognostic value of hs-cTn is unclear.

In the Multi-Ethnic Study of Atherosclerosis (MESA), hs-TnT and NT-proBNP could identify the subset of patients with LV hypertrophy at a higher risk for incident HF, both with and without impaired systolic function [60,61]. In a retrospective analysis of the longitudinal The St Vincent’s Screening TO Prevent Heart Failure (STOP-HF) study, hs-TnI at baseline was a significant predictor of HFpEF development among individuals with risk factors for HF, whereas changes in plasma levels over time were not predictive [62]. The hs-Tn thresholds for risk stratification in the general population have been recently suggested: hs-TnI < 4 or <6 ng/L is indicative of low risk in women and men, respectively, and >10 or >12 ng/L is indicative of a higher risk [63,64,65].

Several studies investigated the prognostic value of troponin assay in patients hospitalized for HF and having a preserved EF. In a cohort of 500 patients with LVEF ≥ 40%, TnT was directly correlated with serum creatinine and symptom severity, and independently predicted all-cause death and HF rehospitalization [66]. In a recent study enrolling 847 HF patients (43% with HFpEF), the AUC of hs-TnT for the prediction of mortality at 30 days was significantly lower in patients with HFpEF (AUC 0.61) than in those with HFmrEF (AUC 0.80, *p* = 0.01) or HFrEF (AUC 0.74, *p* = 0.04). hs-TnT displayed no significant association with 30-day outcome in the HFpEF group (odds ratio [OR], 1.48; [95%-CI 0.89–2.46]; *p* = 0.13), as opposed to HFmrEF (OR 4.53 [95%-CI 1.85–11.1]; *p* < 0.001) and HFrEF (OR 2.58 [95%-CI 1.57–4.23]; *p* < 0.001), suggesting a lesser prognostic value of hs-TnT in HFpEF [67]. As for chronic HF, a study on 155 HF patients (41% with HFpEF) over a median follow up of 449 days revealed that patients with HFpEF who developed adverse events had higher hs-TnT concentrations compared to those who did not (36 (20–66) ng/L vs. 21 (15–32) ng/L, *p* = 0.003). The AUC for hs-TnT was higher than BNP (0.739 vs. 0.631), and the optimal hs-TnT cut-off for adverse events (all-cause mortality, non-fatal myocardial infarction, non-fatal stroke, HF hospitalization) was 26 ng/L [58].

The phase 2 Prospective comparison of ARNI (angiotensin receptor–neprilysin inhibitor) with ARB (angiotensin-receptor blockers) on Management Of heart failUre with preserved ejectioN fraction (PARAMOUNT) and the phase 3 Prospective Comparison of ARNI with ARB Global Outcomes in HF With Preserved Ejection Fraction (PARAGON-HF) trials randomized patients with HFpEF to sacubitril/valsartan or valsartan, showing that patients receiving sacubitril/valsartan had a greater reduction in hs-TnT compared to those assigned to valsartan (PARAMOUNT: 14%, *p* = 0.03; PARAGON-HF: 9%, *p* < 0.001) [68,69]. Moreover, in the PARAGON-HF trial, patients with hs-TnT levels reduced by 16 weeks to ≤17 ng/L (median value at baseline) had a lower risk of CV death or HF hospitalization compared with those with persistently elevated hsTnT (*p* = 0.046) [69].

Data regarding the prognostic ability of troponins also derive from a recent analysis of the EMPEROR-Preserved trial. Of 5988 study participants with LVEF > 40%, 5825 (97.3%) had available hs-TnT measurements, with an overall median baseline of 17.8 ng/L (Q1 and Q3 at 11.6 and 26.9 ng/L respectively) and 3767 (65.7%) patients showing hs-TnT > 14 ng/L [37]. Similar to the results for NT-proBNP concentrations, higher hs-TnT levels were associated with more severe HF and comorbidities. A significant increase in the rates of CV death and HF hospitalization was observed across quartiles, with a 5-fold higher number of events between quartiles 1 and 4 in patients randomized to placebo [37]. Similar results were obtained in another analysis of EMPEROR-Preserved trial, in which patients with both the lowest NT-proBNP and lowest hs-TnT had a primary event (CV death and HF hospitalization) rate of 2.2 per 100 patient-years compared to 19.2 per 100 patient-years in those with highest NT-proBNP and hs-TnT, with a rate ratio of 8.7 [70]. Finally, treatment with empagliflozin resulted in a beneficial impact on cardiac outcomes independently from the baseline NT-proBNP and hs-TnT levels evaluated [37].

## 4. Biomarkers in HFpEF beyond Natriuretic Peptides and Troponin

Different circulating biomarker profiles have emerged in patients with LVEF ≤ 40% or LVEF ≥ 50%. Patients with HFpEF display significantly higher levels of soluble suppression of tumorigenesis 2 (sST2), hs-C-reactive protein (CRP) and cystatin-C, while HFrEF patients presented higher concentrations of NT-proBNP, hs-TnT and hemoglobin [57]. Moreover, emerging molecules such as tissue inhibitor of metalloproteinases (TIMPs), N-terminal propeptide of type III procollagen (PIIINP), homocysteine and resistin are particularly upregulated in HFpEF compared with HFrEF; while biomarkers of inflammation, such as pentraxin-3, tumour necrosis factor alpha (TNF-a), interleukin (IL)-6 and IL-10, show increased levels in HFrEF compared with HFpEF [71]. The biomarkers that have been evaluated most extensively in HFpEF are sST2, galectin-3 (Gal-3), growth differentiation factor 15 (GDF-15), and some other inflammatory biomarkers.

### 4.1. sST2

sST2 is a member of the Toll-like/interleukin-1 receptor superfamily, which is expressed following myocardial stretch and cardiovascular injury, together with its ligand, IL-33. The IL-33/ST2 interaction exerts anti-apoptotic, anti-hypertrophic and anti-fibrotic actions [72,73], while the soluble form of ST2 (sST2), produced by alternative splicing [74], acts as a decoy receptor, blunting the beneficial effects of the IL-33/ST2 axis. sST2 is mainly synthesized in extracardiac sites in response to hemodynamic overload, inflammation and profibrotic stimuli [75]. In HFpEF, an association has been reported between sST2 levels and proinflammatory comorbidities, right ventricular pressure overload, and systemic congestion, but not with LV geometry or function [76].

Santhanakrishnan et al. reported that sST2 was not significantly higher in patients with HFpEF (*n* = 50) than in sex-matched controls aged ≥55 years without HF or coronary artery disease (*n* = 50) [56]. Conversely, Wang et al. compared patients with hypertension and HFpEF (*n* = 68, 64%) to a control group with hypertension without HF (*n* = 39, 36%), finding that sST2 was higher in HFpEF than controls (*p* < 0.001), although patients with HFpEF were also older [77]. Even Sinning et al., who compared HFpEF patients (*n* = 70) with controls without HF (*n* = 4864), found that the median sST2 level was higher in HFpEF (26.5 ng/mL; interquartile range [IQR]: 21.7–36.0 ng/mL) compared to controls (24.5 ng/mL; IQR: 20.1–30.7 ng/mL) [78]. Overall, the differences in circulating sST2 between HFpEF patients and controls are small, possibly also because sST2 is not a cardiac-specific biomarker. In the RELAX trial, which randomized 216 HFpEF patients 1:1 to sildenafil vs. placebo, higher sST2 levels were associated with the presence of several proinflammatory comorbidities: diabetes (*p* = 0.049), hypertension (*p* = 0.023), AF (*p* = 0.049), renal dysfunction (*p* < 0.001) and congestion (*p* < 0.001) [76].

sST2 has emerged as a powerful predictor of outcome in chronic HF, and has been evaluated specifically in the setting of HFpEF [79,80]. In a study conducted on 458 HFrEF and 112 HFpEF patients from the TIME-CHF trial, sST2 showed an association with overall survival at 18-month in patients with HFpEF (HR: 12.18, 95% CI 2.45–60.65). HFpEF patients also exhibited higher sST2 levels compared with HFrEF patients (37.6 ng/mL, 95% CI 28.5–54.7 vs. 35.7 ng/mL, 95% CI 25.6–52.2, *p* = 0.02) after adjustment for clinical covariates (age, sex, BMI, systolic blood pressure, AF) [57].

In a cohort of 191 patients with acute decompensated HFpEF, sST2 emerged as a significant predictor of non-cardiovascular mortality (HR 1.03, 95% CI 1.00–1.04), during a median follow-up of 445 days. Indeed, sST2 levels were increased in patients with all-cause death and non-cardiovascular death compared to those without events (median of 23.1 ng/mL vs. 17.1 ng/mL for all-cause death, *p* = 0.004; 36.6 ng/mL vs. 17.9 ng/mL for non- cardiovascular death, *p* = 0.02) [81]. Similar results were obtained in a study population of 193 (HFpEF *n* = 86; HFrEF *n* = 86, control without HF *n* = 21) including acute decompensated HF subjects. In both crude analyses (HR per log increase 10.04; 95% CI 1.89–53.44) and adjusted model (HR 6.62; 95% CI 1.04–42.28) of HFpEF, sST2 was significantly associated with death from any cause or hospitalization for HF [82]. Moreover, sST2, but not NT-proBNP, was identified as an independent predictor of intermediate-term mortality in 200 HFpEF patients with acute dyspnea [83].

### 4.2. Gal-3

Galectin-3 (Gal-3) is a soluble β-galactoside binding lectin produced by macrophages, which plays an important role in the development of cardiovascular damage and remodeling by promoting the processes of cardiovascular inflammation, fibroblast proliferation and fibrosis, which drive the progression from subclinical structural cardiac involvement toward the development of HFpEF.

Gal-3 levels in plasma are increased in patients with HFpEF compared to controls [84]. Gal-3 also shows a correlation with indices of diastolic dysfunction, LV stiffness and severity of HFpEF [85,86,87]. In a case–control study including 63 HFpEF cases and 20 age- and sex-matched healthy controls, Gal-3 had an AUC of 0.927 to diagnose HFpEF, while NT-proBNP had an AUC of 0.871. A Gal-3 cut-off of 10.1 ng/mL had a 78% sensitivity and a 95% specificity to diagnose HFpEF, with a positive predictive value of 98% and a negative predictive value of 59% [88]. In a study on 247 patients (172 classified as HFpEF, 30 controls without HF, 45 with HFrEF) followed for 1 year, Gal-3 distinguished HFpEF from controls (AUC = 0.819, 95% CI 0.75–0.89) and HFrEF (AUC = 0.863, 95% CI 0.79, 0.93), while sST2 had a lower AUC for predicting HFpEF vs. controls (AUC = 0.584, 95% CI 0.49–0.68) and HFpEF vs. HFrEF (AUC = 0.824, 95% CI 0.73–0.90), revealing that Gal-3 seemed superior to sST2 in distinguishing HFpEF from controls and HFrEF [89]. The observational study on Prevalence and Clinical Course of Diastolic Dysfunction and Diastolic Heart Failure (Diast-CHF) enrolled 1386 subjects with >1 cardiovascular risk factor (170 with HFpEF). Gal-3 had an AUC of 0.71 (while NT-proBNP had an AUC of 0.59), and a Gal-3 cut-off of 14 ng/mL had 61% sensitivity and 73% specificity to diagnose HFpEF [90].

Gal-3 also predicted long-term outcome in HFpEF patients [90,91,92,93]. Notably, the predictive value of Gal-3 seems greater in HFpEF than in HFrEF, as shown by a sub-analysis of data from the The Coordinating study evaluating Outcomes of Advising and Counseling in Heart Failure (COACH) study, where plasma Gal-3 was associated with a higher risk for death and HF hospitalization (patients in the second quartile had HR = 1.98, 95% CI 1.29–3.02; patients in the third quartile a HR = 2.66, 95% CI 1.76–4.03, patients in the fourth quartile a HR = 3.34, 95% CI 2.23–5.01) in 592 HF patients (HFpEF *n* = 107; HFrEF *n* = 485) followed up for 18 months, but a similar elevation in plasma Gal-3 levels represents a much higher risk for all-cause death and hospitalization due to HF in patients with HFpEF vs. HFrEF (*p* < 0.001) [91]. In a cohort of 419 HF patients with LVEF > 45%, plasma Gal-3 above 14 ng/mL was a significant predictor of all-cause mortality and HF hospitalization at 1 year (adjusted HR: 1.43, 95% CI 1.07–1.91) [94].

A study on 247 patients (70% with HFpEF) reported that Gal-3 was strongly correlated with an increased risk of CV death and HF hospitalization in HFpEF patients, and the HR was 2.33 per 1 standard deviation increase in Gal-3 (95% CI: 1.72–2.94, *p* = 0.009) after adjustment for clinical factors and NT-proBNP [89]. Moreover, in a population of 592 HF patients, Gal-3 concentration was a stronger predictor of mortality for patients with HFpEF than in those with HFrEF [91]. However, the prognostic value of Gal-3 in HFpEF and HFrEF is lower than other molecules such as NT-proBNP or sST2, and largely depending on renal function [95]. An analysis of the Aldo-DHF trial evaluated the association between spironolactone treatment and Gal-3 concentrations over time, and the correlation between Gal-3 changes and clinical outcomes. Increased baseline Gal-3 (>12.1 ng/mL) levels at 6 or 12 months were associated with higher risk of all-cause mortality and hospitalization independently of NT-proBNP (adjusted HR: 3.127, 95% CI 1.144–8.549) [92].

### 4.3. GDF-15

GDF-15 is a member of the transforming growth factor-β (TGF-β) cytokine superfamily. It is synthesized as an inactive precursor that undergoes proteolytic activation, releasing the N-terminal pro-peptide from the mature GDF-15 protein, which is then secreted as a dimer [96,97]. GDF-15 is released in response to inflammation, oxidative stress, hypoxia, telomere erosion, and oncogene activation, and exerts anti-inflammatory and anti-apoptotic actions [98]. In physiological conditions, GDF-15 is weakly expressed in human tissues, except for the placenta, while it can be produced by many cardiovascular and non-cardiovascular cells under pathological conditions. GDF-15 is usually upregulated in acute myocardial infarction, inflammation, and cancer [99]. Differently from NT-proBNP, GDF-15 levels are not influenced by AF [100].

Circulating GDF-15 levels are higher in patients with HFpEF compared to controls [56,78,101,102]. In a subgroup of patients from the DIAST-CHF study, patients with HFpEF (*n* = 142) showed higher GDF-15 levels than controls (*n* = 188): 1.66 ng/mL (IQR:1.26–2.34 ng/mL) vs. 0.9 ng/mL (IQR: 0.70–1.09 ng/mL) (*p* < 0.001), respectively [102]. Dinh et al. evaluated that GDF-15 levels were increased in patients with HFpEF compared to controls and were also higher in subjects with moderate to severe compared to mild diastolic dysfunction [101]. The combination of GDF-15 and NT-proBNP did not improve the diagnostic performance over NT-proBNP alone [56]. The NT-proBNP/GDF-15 ratio has been shown to discriminate between HFpEF and HFrEF (AUC 0.709) [56]. Nonetheless, in a population of 311 patients followed up for 15 months (29% with HFmrEF and 71% with HFpEF), no differences in GDF-15 or NT-proBNP levels were identified between both groups. In clinical studies, the median GDF-15 values were comparable in HFpEF and HFrEF [103].

The increase in plasma GDF-15 seems to hold prognostic significance in HF [104]. In a cohort of 380 patients, GDF-15, Gal-3, and sST2 were able to predict the 2-year risk of death in patients with acute HFpEF [105]. In the study above with 29% of patients with HFmrEF and 71% with HFpEF, those who died had higher median concentrations of GDF-15 (4085 vs. 2270 ng/L, *p* < 0.001) and NT-proBNP (1984 vs. 1095 ng/L, *p* < 0.001) [106].

### 4.4. Purely Inflammatory Biomarkers

Systemic inflammation is a crucial determinant of HFpEF development [107], but only scarce evidence exists on the clinical utility of inflammatory biomarkers. A metanalysis including 19 studies (*n* = 51,196) revealed a significant association between high CRP levels and new-onset HFpEF (HR 1.08; 95% CI: 1.01–1.16; *p* = 0.04) [108]. A case-cohort study based on the Prevention of Renal and Vascular End-Stage Disease (PREVEND) trial, which enrolled 961 participants without HF at baseline, followed up for a median of 8.2 years, reported that IL-6 was significantly associated with new-onset HFpEF independently of confounders (e.g., sex, age, body mass index, eGFR, hypertension, diabetes, etc.) (HR 1.59; 95% CI, 1.16–2.19; *p* = 0.004), but not with HFrEF (HR 1.28; 95% CI, 1.02–1.61; *p* = 0.03) [109]. In a study conducted on 6814 patients over 10.9 years, CRP and IL-6 were both associated with an increased risk of HFpEF development (HR 1.21 per doubling; 95% CI: 1.01 to 1.45, and HR 1.51 per doubling; 95% CI 1.09 to 2.10, respectively) but not with HFmrEF or HFrEF [110].

Macrophage migration inhibitory factor (MIF) has recently attracted some attention. MIF is a pleiotropic mediator of the innate immune system, which induces the release of several cytokines, including TNF-α which activates matrix metalloproteinases and triggers collagen degradation [111,112]. In a cohort of 62 patients with HFpEF followed for 6 months, the median MIF level was 51.6 ng/mL (IQR 35.6 ng/mL, range 6.4–168.6). Patients with increased MIF concentrations were more likely to have a worse NYHA functional class (*p* < 0.001) and a higher pulmonary artery systolic pressure (*p* = 0.002) [112]. MIF levels displayed an association with all-cause death or hospitalization (adjusted HR 2.35, 95% CI 1.05–5.27, *p* = 0.037). The predictive value of MIF for the occurrence of death or hospitalization at 180 days was inferior to NPs (BNP: AUC = 0.66, *p* = 0.027; NT-proBNP: AUC = 0.68, *p* = 0.017) [112].

## 5. Conclusions and Future Perspectives

HFpEF is a heterogeneous clinical syndrome, with different cardiovascular and non-cardiovascular determinants participating in its development and presentation. Many biomarkers have been tested as possible tools for the diagnosis and management of HFpEF in the past years, only rarely showing promising results. B-type NPs have an established diagnostic role, and both B-type NPs and hs-Tn may help predict future disease trajectories. The notion of HFpEF as a systemic disorder has led to the assessment of multiple biomarkers exploring systemic inflammation, fibrosis or multiple mechanisms (as in the case of sST2 and GDF-15). Non-cardiac-specific biomarkers have no diagnostic role but may refine risk prediction in patients with established HFpEF, or signal an increased risk of HFpEF in patients with predisposing conditions. Biomarkers could also be measured serially over time, to gain insight on disease evolution and the response to treatment. Furthermore, the combination of multiple biomarkers might allow to investigate different disease pathways.

The development of molecular biology techniques provides the opportunity to investigate and increase our understanding of HFpEF. Proteomics allows the identification and quantification of all proteins of a cell, tissue or an organism [113]. The advent of proteomic techniques such as targeted mass spectrometry, DNA aptamer and antibody-based approaches has provided researchers with innovative ways to manage the health status of patients, but also to identify the dynamic protein changes which reflect the peculiar fluctuations in HF status and the impact of treatment interventions [113,114]. In a study conducted on 173 HF patients, 120 proteins (62 increased, 58 decreased) were differentially expressed in patients with HFrEF (*n* = 47) compared with HFpEF (*n* = 43), whereas 54 proteins (31 increased, 23 decreased) were differentially expressed in patients with HFmrEF (*n* = 83) compared with HFpEF [115].

A proteomic approach enables us to achieve a deeper understanding of the pathophysiological pathways of diseases, as showed by the proteomic identification of quiescin Q6, a protein involved in the formation of disulphide bonds, which allows to discriminate acute HF from non-cardiac dyspnea [116]. Moreover, innovative proteomic strategies aim at defining the “proteomic signatures” unique to a certain HF phenotype, such as HFpEF, HFrEF, and HFmrEF [115]. These signatures may also help stratify patient prognosis.

Some Authors have also performed -omic profiling to find novel biomarkers associated with incident HF or HF prognosis [113]. For example, plasma long-chain acylcarnitine concentration was significantly increased in HF patients compared to controls, and even discriminated HFrEF from HFpEF, being higher in HFrEF [117]. Moreover, machine-learning algorithms applied to a large number of biomarkers in HFpEF populations has identified clusters with different phenotypes and clinical course [118]. Multi-marker panels and the -omic techniques definitely offer engaging new perspectives for future research.

## Data Availability

Not applicable.
